# Post-COVID dyspnea: prevalence, predictors, and outcomes in a longitudinal, prospective cohort

**DOI:** 10.1186/s12890-023-02376-w

**Published:** 2023-03-13

**Authors:** Japnam S. Grewal, Christopher Carlsten, James C. Johnston, Aditi S. Shah, Alyson W. Wong, Christopher J. Ryerson

**Affiliations:** 1 Department of Medicine, University of British Columbia, Vancouver, Canada; 2 Centre for Heart Lung Innovation, St. Paul’s Hospital, Vancouver, BC Canada

**Keywords:** COVID-19, Dyspnea, Patient, Outcomes, Predictors

## Abstract

**Background:**

The pathophysiology, evolution, and associated outcomes of post-COVID dyspnea remain unknown. The aim of this study was to determine the prevalence, severity, and predictors of dyspnea 12 months following hospitalization for COVID-19, and to describe the respiratory, cardiac, and patient-reported outcomes in patients with post-COVID dyspnea.

**Methods:**

We enrolled a prospective cohort of all adult patients admitted to 2 academic hospitals in Vancouver, Canada with PCR-confirmed SARS-CoV-2 during the first wave of COVID between March and June 2020. Dyspnea was measured 3, 6, and 12 months after initial symptom onset using the University of California San Diego Shortness of Breath Questionnaire.

**Results:**

A total of 76 patients were included. Clinically meaningful dyspnea (baseline score > 10 points) was present in 49% of patients at 3 months and 46% at 12 months following COVID-19. Between 3 and 12 months post-COVID-19, 24% patients had a clinically meaningful worsening in their dyspnea, 49% had no meaningful change, and 28% had a clinically meaningful improvement in their dyspnea. There was worse sleep, mood, quality of life, and frailty in patients with clinically meaningful dyspnea at 12 months post-COVID infection compared to patients without dyspnea. There was no difference in PFT findings, troponin, or BNP comparing patients with and without clinically meaningful dyspnea at 12 months. Severity of dyspnea and depressive symptoms at 3 months predicted severity of dyspnea at 12 months.

**Conclusions:**

Post-COVID dyspnea is common, persistent, and negatively impacts quality of life. Mood abnormalities may play a causative role in post-COVID dyspnea in addition to potential cardiorespiratory abnormalities. Dyspnea and depression at initial follow-up predict longer-term post-COVID dyspnea, emphasizing that standardized dyspnea and mood assessment following COVID-19 may identify patients at high risk of post-COVID dyspnea and facilitating early and effective management.

## Background

Dyspnea is a common symptom following COVID-19 and has a significant impact on quality of life [1–3]; however, our understanding of the mechanisms of dyspnea in this specific context also remain limited. Although some patients have cardiopulmonary abnormalities post-COVID, dyspnea can persist in others despite improvements in and normalization of cardiopulmonary function [4–7].

The underlying pathophysiology behind this ‘unexplained’ dyspnea remains unknown, with previous studies showing conflicting results. While some studies showed no difference between healthy controls and patients with post-COVID dyspnea [8], others have found exercise intolerance with evidence of circulatory and breathing pattern abnormalities [9, 10]. There is similarly only limited understanding of the evolution of post-COVID dyspnea and associated outcomes over time, and it is difficult to predict the severity of long-term respiratory symptoms following recovery from the acute phase of COVID-19.

The aim of this study was to determine the prevalence, severity, and predictors of dyspnea at 12 months following hospitalization for COVID-19, and to describe the respiratory, cardiac, and patient-reported outcomes in patients with post-COVID dyspnea. These findings would help patients and clinicians better understand the heterogeneous causes of persistent post-COVID dyspnea such that more appropriate management approaches can be considered depending on the specific clinical scenario.

## Methods

### Study population

We enrolled a prospective cohort of patients from the Post-COVID-19 Respiratory Clinic (PCRC) located at two academic hospitals in Vancouver, Canada [3, 5, 6]. All adult patients admitted to hospital with PCR-confirmed SARS-CoV-2 during the first wave of COVID between March and June 2020 within the Vancouver Coastal Health Authority were automatically referred to the PCRC for follow-up care after hospital discharge. Eligibility criteria included hospitalization for COVID-19, ability to complete study questionnaires in English, and provision of informed consent. There were no exclusion criteria. Research ethics board approval was obtained from the University of British Columbia (#H20-01,239).

### Measurements

Patients were initially assessed 3 months post-symptom onset, with subsequent follow-up occurring at the 6- and 12-month mark. Patients completed a standardized set of questionnaires and investigations at each visit. Questionnaires included the 5-level EuroQoL 5-Dimensions (EQ-5D-5L) [11], Frailty Index [12], University of California San Diego Shortness of Breath (UCSD) Questionnaire [13], Patient Health Questionnaire-9 (PHQ-9) [14], and Pittsburgh Sleep Quality Index (PSQI) [15]. The EQ-5D-5L measures health-related quality of life based on 5 dimensions that include mobility, self-care, usual activities, pain/discomfort, and anxiety/depression. The Frailty Index quantifies frailty based on accumulated health deficits, which can predict mortality and other health outcomes. The UCSD questionnaire is a validated tool for grading the severity of dyspnea in respiratory diseases. Clinically meaningful dyspnea at baseline was defined as a UCSD score > 10, equating to a value that is double the previously established minimal important difference (MID) [16, 17]. The PHQ-9 is a self-administered tool for grading the severity of depression. A mood abnormality was defined as a PHQ-9 score ≥ 5, which has been previously validated as the threshold for mild depression [14]. The PSQI rates sleep quality over a 1-month interval. Higher values on the questionnaires represent worse outcomes except for the EQ-5D-5L.

Standard investigations performed at each clinic visit included standardized bloodwork, detailed pulmonary function tests (PFTs), transthoracic echocardiogram (TTE; only performed at the 3-month visit), and 6-min walk test (6MWT) [5, 6]. PFTs were conducted in accordance with international guidelines, with values < 80% predicted considered abnormal for forced vital capacity (FVC), forced expiratory volume in the first second (FEV1), total lung capacity (TLC), residual volume (RV), and diffusion capacity of carbon monoxide (DLCO) [18–21]. TTEs were conducted in accordance with international guidelines and were interpreted by cardiologists with advanced echocardiography training [22, 23]. A normal left ventricular ejection fraction (LVEF) was defined as ≥ 50% and a normal pulmonary artery systolic pressure (PASP) was defined as less than < 30 mmHg based on international guidelines [22].

### Statistical analysis

Data are shown as mean ± standard deviation, median (interquartile range), or number (percent). Student’s t-tests, Wilcoxon signed rank tests, or Spearman’s rank correlations were used to compare measurements between 3 and 12 months, and dyspneic and non-dyspneic patients, with the choice of test depending upon variable type (dichotomous vs continuous) and variable distribution (for continuous variables). Associations between pre-defined predictor variables and UCSD scores were determined using multivariable linear regression models. Prespecified covariates included age, sex, and body mass index (BMI). Statistical significance was defined as a two-tailed p-value < 0.05, with STATA 16.1 used for data analysis (StataCorp, Texas).

## Results

### Patient characteristics

**Fig. 1:**
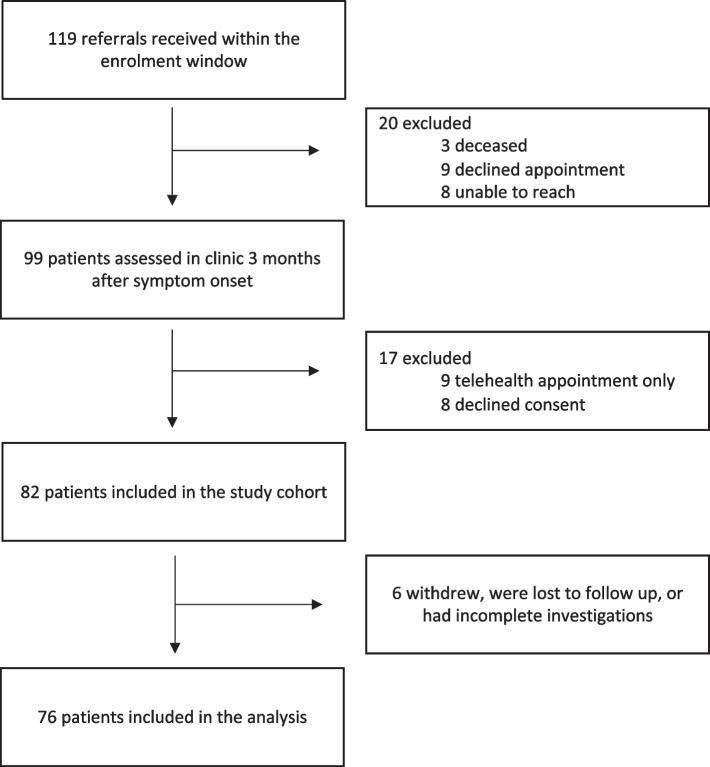
Study enrolment

A total of 76 patients were included (Fig. 1). Pre-existing respiratory disease was present in 29% of patients with clinically meaningful dyspnea at 12 months post-COVID and 15% of patients without dyspnea (Table1). The median length of admission to hospital was 11 (5–17) days in dyspneic patients and 6.5 (5–11) days in non-dyspneic patients. The frequency of critical care admission and mechanical ventilation was similar among patients with and without dyspnea at 12 months post-COVID. Median length of supplemental oxygen requirement was 11 (7–19) days in dyspneic patients, compared to 5 (3–11) in non-dyspneic patients.

**Table 1:** Patient characteristics

	**No dyspnea (*n* = 41)**	**Dyspnea (*n* = 35)**
**Demographics**		
*Age*	60 ± 16	63 ± 16
*Male*	27 (66)	20 (57)
*Ever smoker*	10 (25)	13 (37)
**Comorbidities**		
*Cardiac*	4 (10)	10 (29)
*Pulmonary*	6 (15)	10 (29)
*Renal*	2 (5)	5 (14)
*Diabetes*	11 (27)	7 (20)
*Hypertension*	15 (37)	11 (31)
*Dyslipidemia*	12 (29)	12 (34)
*Malignancy*	4 (10)	4 (11)
**COVID-19 infection characteristics**		
*Admission to hospital*	40 (98)	34 (97)
*Length of admission in days*	6.5 (5–11)	11 (5–17)
*Length of oxygen requirements in days*	5 (3–11)	11 (7–19)
*ICU/HAU admission*	19 (46)	17 (49)
*Mechanical ventilation*	7 (17)	6 (17)

Data shown are mean ± standard deviation, median (interquartile range), or number (%). Dyspnea was defined as a UCSD score > 10 at 12 months post-COVID-19. Pulmonary comorbidity was defined as any formally diagnosed lung parenchymal, pulmonary vascular, pleural, airway, or sleep-related abnormality. Cardiac comorbidity was defined as any formally diagnosed coronary artery disease, valvular abnormality, heart failure, cardiomyopathy, or arrythmia. Malignancy was defined as any formally diagnosed malignant tumour

### Dyspnea at 12 months post-COVID-19 infection

**Fig. 2:**
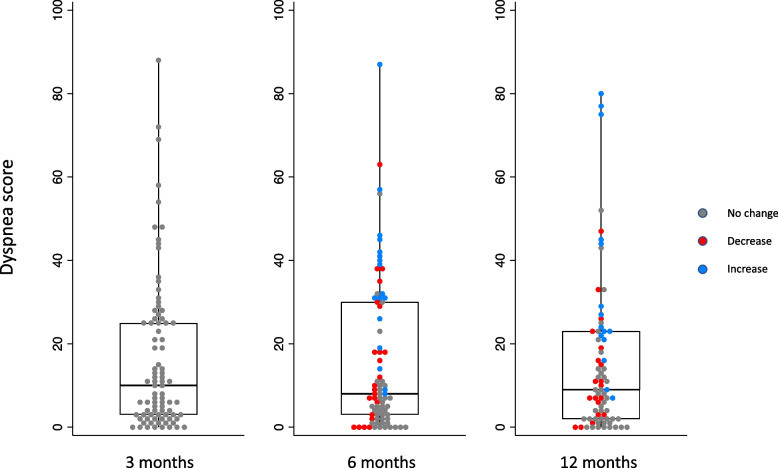
Dyspnea over time following COVID-19. A clinically meaningful change in dyspnea was defined as a change in UCSD score equal to or greater than 5, based on the previously established MCID for the UCSD questionnaire. Change in dyspnea, as represented by the different colours of dots, is relative to the dyspnea score at 3 months post-COVID-19

Evolution of dyspnea scores over time is shown in Fig. 2. Clinically meaningful dyspnea was present in 37 (49%) of patients at 3 months and 35 (46%) of patients at 12 months following COVID-19, with median UCSD scores of 10 (3–25) and 9 (2–23) respectively (*p* = 0.54). Of patients who were dyspneic at 12 months, 28 (80%) had dyspnea that had persisted from their 3-month visit, and 7 (20%) had new-onset of clinically meaningful dyspnea. Between 3 and 12 months post-COVID-19, 18 (24%) patients had a clinically meaningful worsening in their dyspnea, 37 (49%) had no meaningful change, and 21 (28%) of patients had a clinically meaningful improvement in their dyspnea. There was no significant difference in the severity of dyspnea comparing patients who did and did not require mechanical ventilation or comparing those requiring and not requiring intensive care unit (ICU) admission.

### Association of dyspnea with other patient-reported, respiratory, and cardiac outcomes

**Fig. 3:**
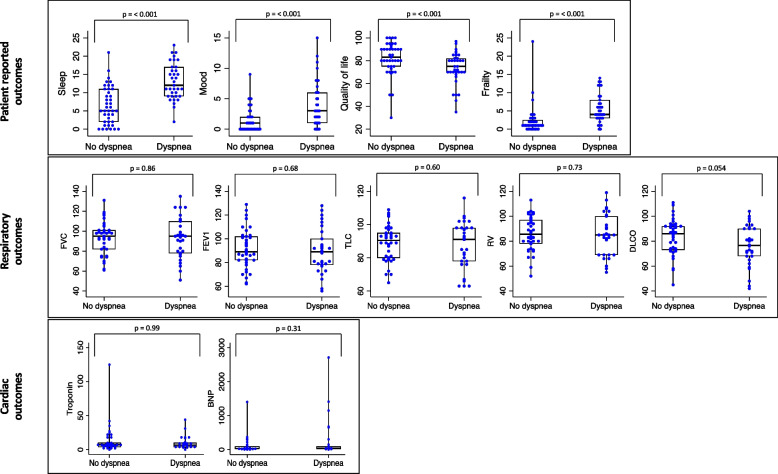
Patient-reported, respiratory, and cardiac outcomes 12 months post-COVID-19 stratified by presence and absence of dyspnea. Abbreviations: FVC, forced vital capacity; FEV1, forced expiratory volume in the first second; TLC, total lung capacity; RV, residual volume; DLCO, diffusing capacity for carbon monoxide; BNP, B-type natriuretic peptide

There was worse sleep, mood, quality of life, and frailty in patients with clinically meaningful dyspnea at 12 months post-COVID infection compared to patients without dyspnea (Fig. 3). There was no statistically significant difference in PFT findings comparing patients with and without clinically meaningful dyspnea at 12 months post-COVID-19 infection (Fig. 3). However, patients with dyspnea had a lower percent-predicted 6-min walk distance (91 ± 15% vs 102 ± 16%; *p* = 0.01) compared to patients without dyspnea. There was no significant difference in the troponin or B-type natriuretic peptide (BNP) levels of patients with and without dyspnea at 12 months post-COVID-19 infection.

**Fig. 4:**
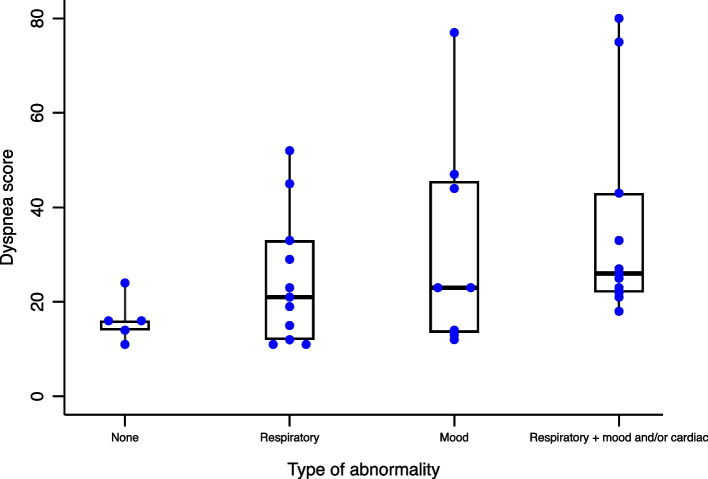
Respiratory, cardiac, and psychological explanations for clinically significant dyspnea 12 months post-COVID-19. No patients had an isolated cardiac abnormality

Of the 35 patients with clinically meaningful dyspnea at 12 months, 22 (63%) had PFT abnormalities, 7 (20%) had abnormal troponin or BNP levels, 13 (37%) had an abnormal depression score, and 5 (14%) had none of these findings. The mean dyspnea score of dyspneic patients with only a PFT abnormality was 25, compared to a mean score of 32 for dyspneic patients with only a mood abnormality (Fig. 4). Patients with multiple abnormalities had a mean dyspnea score of 36. The most common PFT abnormality in dyspneic patients was a decreased DLCO, which was present in 16 patients (46% of those with dyspnea).

### Predictors of dyspnea at 12 months post-COVID-19 infection

The severity of dyspnea and depressive symptoms at 3 months post-COVID-19 infection predicted severity of dyspnea at 12 months post-COVID (Table 2). Neither PFT nor cardiac findings at 3 months predicted dyspnea severity at 12 months. Sensitivity analysis excluding patients with pre-existing respiratory and cardiac comorbidities showed similar findings.

**Table 2:** Associations of 12-month dyspnea score with 3-month patient-reported, respiratory, and cardiac outcomes

Predictor (at 3 months)	Coefficient	P-value
**Patient-reported outcomes**		
UCSD	0.55	< 0.001
PHQ-9	0.36	0.001
**Pulmonary function outcomes**		
FVC	-0.18	0.14
FEV1	-0.19	0.11
TLC	-0.13	0.28
DLCO	-0.21	0.08
6MWD	-0.13	0.31
**Cardiac outcomes**		
BNP	0.15	0.21
D-dimer	0.23	0.06
LVEF	0.16	0.19
PASP	0.18	0.27

Associations between the pre-defined predictor variables and 12-month UCSD scores were determined using multivariable linear regression models. Prespecified covariates included age, sex, and BMI

*Abbreviations*: *UCSD* University of california san diego shortness of breath questionnaire, *PHQ-9* Patient health questionnaire-9, *FVC* Forced vital capacity, *FEV1* Forced expiratory volume in the first second, *TLC* Total lung capacity, *DLCO* Diffusing capacity for carbon monoxide, *6MWD* 6-min walk distance, *BNP* B-type natriuretic peptide, *LVEF* Left ventricular ejection fraction, *PASP* Pulmonary artery systolic pressure

## Discussion

This prospective cohort shows that dyspnea is a frequent symptom following COVID-19, and that most patients with dyspnea do not experience a meaningful improvement in the severity of their symptoms in the first year following infection. We also found that dyspnea was associated with worse sleep, mood, quality of life, and frailty in patients at 12 months post-COVID; however, there was no statistically significant difference in pulmonary function comparing dyspneic and non-dyspneic patients post-COVID. The severity of dyspnea and depressive symptoms at 3 months post-COVID were the only predictors of the severity of dyspnea at 12 months post-COVID. Together, these findings highlight the multifaceted nature of post-COVID dyspnea, with many patients having ongoing dyspnea for reasons other than overt pulmonary or cardiac consequences of COVID-19.

Mood may play a role in post-COVID dyspnea and can predict which patients are at risk for significant persistent dyspnea at 12 months. However, it is unclear whether dyspnea itself is driving these mood abnormalities, or whether the mood abnormality is instead contributing to the development of dyspnea. PTSD as a result of being hospitalized with COVID during a global pandemic may also be playing a role, as it is highly prevalent and associated with more persistent physical symptoms post-COVID [24]. There is a trend towards DLCO being reduced in patients with significant dyspnea at 12 months post-COVID, suggesting a possible underlying persistent cardiopulmonary abnormality that may be contributing to the development of dyspnea in some patients. There is also heterogeneity in the dyspneic patients as some have solely reduced DLCO, while others have primarily mood abnormalities or combinations of PFT, cardiac, and mood abnormalities. Our findings emphasize that dyspnea in the post-COVID context is a complex sensory experience that may be influenced by mood in addition to possible underlying cardiopulmonary pathology, and there may be additional factors that are contributing. Previous studies have established decreased peripheral oxygen delivery and abnormal ventilatory response to aerobic activity as mechanisms of post-COVID dyspnea in the absence of cardiopulmonary limitations on invasive cardiopulmonary exercise testing [9, 10]. Furthermore, based on our findings, the degree of contribution from these factors and the resulting severity of dyspnea appears to differ from patient to patient.

Post-COVID dyspnea was a persistent problem in our patient cohort, with 49% of patients hospitalized for acute COVID reporting no change in their dyspnea, 24% reporting an increase in their dyspnea, and 20% developing new-onset of clinically meaningful dyspnea at the 12-month mark when compared to 3 months post-COVID. The reason for the increase and new onset of dyspnea at the 12-month mark in a subset of patients is unclear but given the similar PFT findings in dyspneic and non-dyspneic patients, other suggested mechanisms may be implicated including changes in mood, peripheral oxygen delivery, and ventilatory response to aerobic activity over time [9, 10, 24]. Furthermore, the morbidity associated with the dyspnea is also persistent, as evidenced by poorer outcomes in all patient-reported variables in those who had persistent dyspnea at 12 months. Interestingly, the severity of the acute infection does not appear to influence the degree of post-COVID dyspnea, as neither the need for ICU admission or for mechanical ventilation were associated with higher dyspnea scores. Previous studies on long term outcomes in patients with acute respiratory distress syndrome (ARDS) from various etiologies have also demonstrated similar prevalence of dyspnea and mood abnormalities as well as reduced DLCO at 12 months post-ARDS, but the study populations entirely consisted of patients who required mechanical ventilation [25, 26]. We demonstrated similar long-term findings despite 82% of our patients not requiring mechanical ventilation and 51% not requiring ICU admission. This emphasizes the need to use standardized dyspnea and depression assessment tools, rather than traditional indicators like severity of illness or level of oxygen requirements, to identify patients who are at higher risk of post-COVID dyspnea given the multiple determinants of dyspnea beyond the degree of lung injury and resultant pulmonary pathology.

The persistent dyspnea and associated morbidity suggests potential benefit of using a validated dyspnea questionnaire at post-COVID follow-up visits, with similar rationale supporting utility of a standardized mood questionnaire. These can be applied in a variety of settings, including primary care clinics to both identify abnormalities and follow change over time, including potential response to intervention. Dyspnea and mood questionnaires could also be used at the time of discharge from hospital to determine who would benefit from outpatient follow-up and resources. Patients identified to have post-COVID dyspnea may benefit from early referral to supportive counselling and other psychiatric resources given the association of mood abnormalities with dyspnea. As well, dyspneic patients may also benefit from referral to pulmonary rehab, as previous literature demonstrates improvement in dyspnea and mood in the post-COVID patient population [27].

Limitations of our study include the lack of baseline characteristics for the patients prior to the initial COVID-19 diagnosis. We attempted to account for any baseline cardiopulmonary abnormalities by performing a sensitivity analysis that excluded patients with known pre-existing cardiac or pulmonary disease, which yielded consistent findings to the analysis based on all included patients. Another limitation of our study is that newer variants of SARS‑CoV‑2 may result in varying prevalence and severity of post-COVID dyspnea compared to our study cohort compared to the α variant that was responsible for wave 1 infections. Vaccination may also influence the prevalence and severity of post-COVID dyspnea [28, 29], which was not available at the time of initial infection. Thus, our results may not be generalizable to all post-COVID patient populations. Repeating this study in vaccinated and non-hospitalized post-COVID patients would help characterize post-COVID dyspnea in these populations.

In summary, post-COVID dyspnea is common, persistent, and has a significant impact on quality of life. Mood abnormalities may play a role in post-COVID dyspnea in addition to potential cardiorespiratory abnormalities. Dyspnea and depression at initial follow-up predict longer-term post-COVID dyspnea, emphasizing the need for standardized dyspnea and mood assessment following COVID-19 to identify patients at higher risk of post-COVID dyspnea and facilitate early and effective management.

## Data Availability

The datasets used and/or analysed during the current study are not openly available as they contain information that could compromise research participant privacy and/or consent. Data may be available from the corresponding author on reasonable request.

## Abbreviations

EQ-5D-5L: 5-Level EuroQoL 5-Dimensions
UCSD: University of California San Diego Shortness of Breath Questionnaire
PHQ-9: Patient Health Questionnaire-9
PSQI: Pittsburgh Sleep Quality Index
MID: Minimal important difference
PFTs: Pulmonary function tests
TTE: Transthoracic echocardiogram
6MWT: 6-Minute walk test
FVC: Forced vital capacity
FEV1: Forced expiratory volume in the first second
TLC: Total lung capacity
RV: Residual volume
DLCO: Diffusion capacity of carbon monoxide
LVEF: Left ventricular ejection fraction
PASP: Pulmonary artery systolic pressure
ICU: Intensive care unit
BNP: B-type natriuretic peptide
ARDS: Acute respiratory distress syndrome
